# Cephalometric measurements and their impact on interlabial distance in individuals with and without anterior open bite: A comparative study

**DOI:** 10.4317/jced.63457

**Published:** 2025-12-30

**Authors:** Leslie Nicole Garcia-Cahuana, Yalil Augusto Rodríguez-Cárdenas, Gustavo Armando Ruíz-Mora, Pedro Luis Tinedo-López, Luis Ernesto Arriola-Guillén

**Affiliations:** 1 Postgraduate student. School of Dentistry, Universidad Científica del Sur, Lima, Perú. ORCID ID: 0000-0003-3580-0414; 2 Ph.D. and Associate Professor of the Division of Oral and Maxillofacial Radiology, School of Dentistry, Universidad Nacional de Colombia, Bogotá, Colombia. ORCID ID: 0000-0002-3107-3013; 3 Ph.D. and Associate Professor of the Division of Orthodontics, Faculty of Dentistry, Universidad Nacional de Colombia, Bogotá D.C, Colombia. ORCID ID: 0000-0002-9954-1047; 4 MSc. and Associate Professor of the Division of Implantology, Universidad Científica del Sur, Lima, Perú. ORCID ID: 0000-0002-2102-4437; 5 Ph.D. and Associate Professor of the Division of Orthodontics, Universidad Científica del Sur, Lima, Perú. ORCID ID: 0000-0003-0010-5948

## Abstract

**Background:**

There has been limited research on the relationship between bone, dentoalveolar structures, and lip sealing, with no comparative studies on individuals with anterior open bite (AOB). This research aimed to evaluate the cephalometric measurements that modify interlabial distance in individuals with and without AOB.

**Material and Methods:**

This retrospective cross-sectional study included 110 cephalometric radiographs (55 with AOB and 55 matched controls). Eighteen cephalometric variables (9 angular and 9 linear) were measured using Blue Sky Plan 4 software (USA) by one trained and calibrated dentist. Data were analysed using SPSS version 26. Shapiro-Wilk test assessed normality; Student's t-test or Mann-Whitney test were applied accordingly. Multiple linear regression analyses were conducted to identify variables that modify interlabial distance (p&lt;0.05).

**Results:**

In the control group, significant influences were identified for several factors: maxillomandibular divergence (B=0.03, p=0.019), upper lip height (B=0.06, p=0.032), upper lip to S-line distance (B=-0.05, p=0.024), and lower lip to S-line distance (B=0.05, p=0.047). In contrast, within the AOB group, only lower facial height had a significant influence (B=0.31, p=0.047). The final multiple linear regression analysis for the whole sample showed that AOB (B=0.703, p=0.009) and lower facial height (B=0.177, p=0.027) significantly affected the interlabial gap.

**Conclusions:**

The interlabial gap in individuals with AOB is mainly influenced by vertical facial dimension, while in controls, maxillomandibular divergence and lip-to-S-line distances play a greater role. Moreover, AOB and increased vertical facial dimension are the most influential factors affecting the interlabial gap, highlighting the need to address both during orthodontic diagnosis and treatment planning.

## Introduction

The interlabial gap, defined as the vertical space between the edges of the lips at rest, is a key clinical parameter for assessing lip competence and the harmony of the lower facial third (1). An increased interlabial gap is often indicative of lip incompetence, which is associated with skeletal and dentoalveolar imbalances (1). From both functional and aesthetic perspectives, a passive lip seal with an interlabial distance equal to or less than 3 mm is considered ideal (2 , 3). Therefore, this measure has become a relevant indicator in orthodontic planning, as it reflects the interaction between bone, dental, and soft tissue structures. One of the most frequently associated conditions with an increased interlabial gap is anterior open bite (AOB) (4). This malocclusion, characterized by a lack of contact between the upper and lower incisors during posterior dental occlusion, poses a significant therapeutic challenge due to its multifactorial origin (5). AOB can negatively impact masticatory function, speech, and facial aesthetics (6). The etiology of AOB is often associated with genetic factors, detrimental oral habits like tongue thrusting or finger sucking, functional alterations, and skeletal discrepancies that affect the development of the maxilla and mandible (7). Cephalometry is a widely utilized tool for analyzing the relationships between craniofacial structures and various malocclusions (8). From a cephalometric standpoint, lip incompetence can be influenced by several skeletal and dentoalveolar factors (4 , 9 - 11). Previous studies have shown that specific skeletal characteristics-such as the inclination of the mandibular plane, anterior facial height, and the relationship between the maxilla and mandible-are linked to lip incompetence and AOB (12 - 14). In particular, a hyperdivergent skeletal pattern and an increased angle between the skull base and the mandible can lead to excessive vertical growth, making it difficult for the lips to seal adequately at rest (15). On the dentoalveolar level, the protrusion of the upper incisors can lead to greater dental exposure, hindering passive lip contact (16 , 17). Additionally, both excessive and insufficient eruptions of the incisors can disrupt the vertical relationship between the lips, adversely affecting their ability to seal without tension (14). Furthermore, excessive molar eruption is associated with AOB, which can increase the vertical dimension and contribute to labial incompetence (13 - 16). Despite the clinical significance of these issues, existing literature has primarily centered on the classification and etiology of AOB,(7) and the analysis of changes following orthodontic or orthognathic treatments (18 - 19). There has been insufficient exploration of the relationship between bone and dentoalveolar structures and lip sealing. Additionally, no comparative studies have been conducted between individuals with and without AOB, which limits the understanding of structural differences between these groups. Therefore, this study aims to evaluate the cephalometric measurements that modify interlabial distance in individuals with and without AOB. Identifying these factors will enhance our understanding of the mechanisms influencing lip incompetence and aid in improving both diagnostic and therapeutic approaches.

## Material and Methods

**Figure 1:**
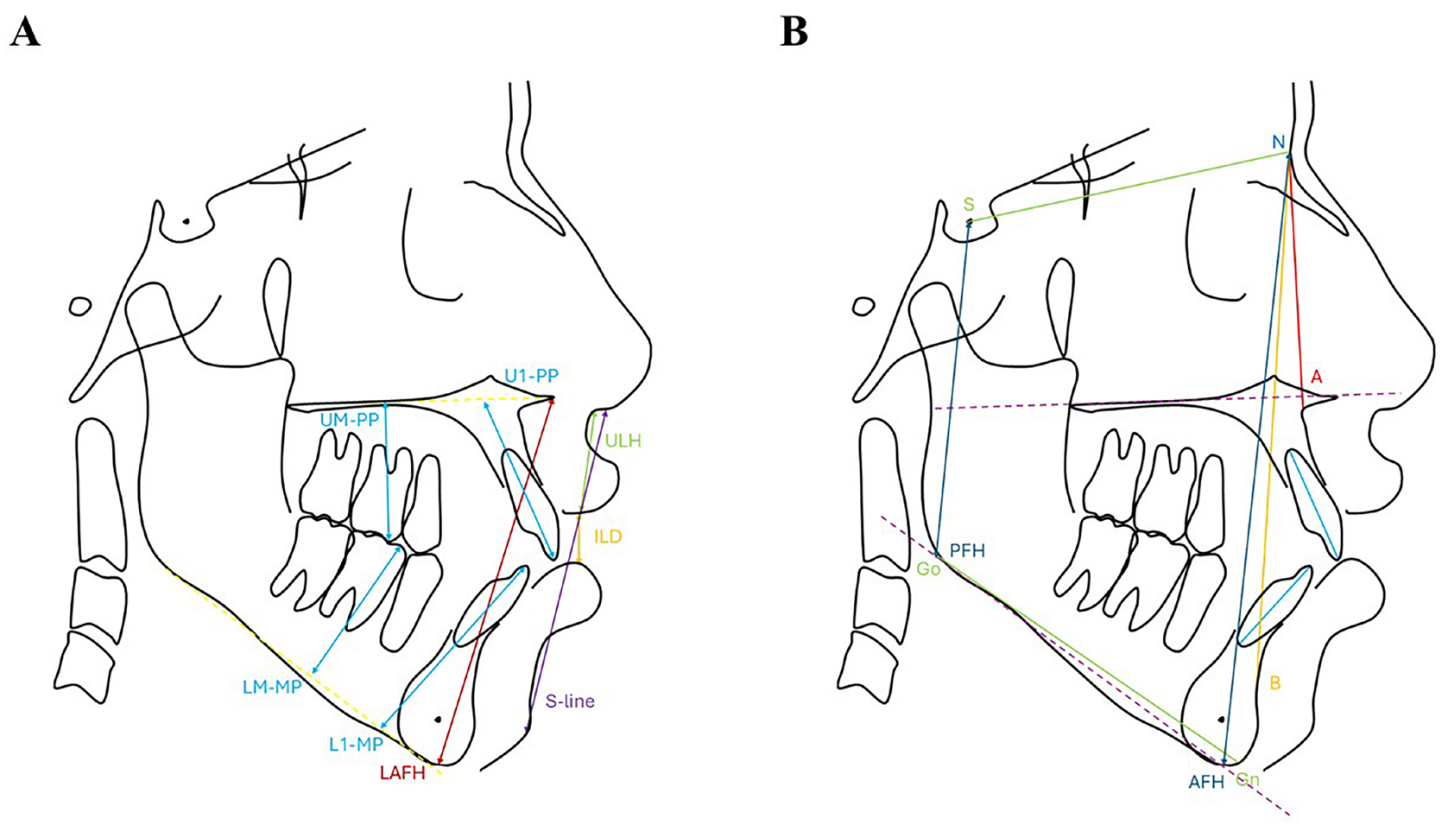
Lineal (A) and angular (B) measurements. LAFH indicates lower anterior facial height; ILD, interlabial distance; ULH, upper lip height; U1-PP, upper incisor to palatal plane; L1-MP, lower incisor to mandibular plane; UM-PP, upper molar to palatal plane; LM-MP, lower molar to mandibular plane; PFH, posterior facial height; AFH, anterior facial height.

- Ethical approval This study was an observational, retrospective, and cross-sectional study that adhered to the Strengthening the Reporting of Observational Studies in Epidemiology (STROBE) guidelines. It received approval from the Institutional Ethics Committee for Research at Universidad Científica del Sur (number 1121-CIEI-CIENTÍFICA-2025). - Basic information One hundred ten cephalometric radiographs were collected while the participants were in the maximum intercuspation position, with their lips at rest and without forcing lip closure. The sample included 55 radiographs from patients diagnosed with AOB, characterized by the absence of contact between the incisal edges of the upper and lower incisors during occlusion, indicating a negative overbite. Additionally, there was a control group consisting of 55 individuals matched for age and sex, for whom radiographs were obtained but who did not have AOB. - Image acquisition Radiographs from patients with a history of orthognathic surgery, previous or ongoing orthodontic treatment, congenital or acquired craniofacial abnormalities, or significant tooth loss were excluded. The sample size was calculated using a formula for estimating two proportions to compare the impact of predictive variables on adequate lip sealing between the AAM and non-AAM groups. A 95% confidence interval (=0.05) and 80% statistical power were set, requiring at least 50 radiographs per group. - Training and calibration Calibration was performed using 30 randomly selected cephalometric radiographs. An examiner, supervised by an orthodontic researcher with over 10 years of experience, conducted the measurements twice-one week apart-to minimize measurement variation. The reliability of the measurements was evaluated using the intraclass correlation coefficient (ICC), with values above 0.85 indicating high reliability. - Variable measurements The cephalometric radiographs were processed and analyzed using BlueSky Plan 4 software (USA) to ensure measurement accuracy. A single, pre-calibrated operator was responsible for tracing and evaluating the parameters to maintain consistency throughout the process. Eighteen cephalometric parameters were analyzed, including nine angular and nine linear measurements. These variables were selected based on their relevance to assessing the relationship between facial structure and lip seal. Table 1 lists the evaluated variables, while Figure 1A,B illustrate the cephalometric parameters.

Table 1

1

- Statistical analyses Descriptive statistics were used to confirm demographic homogeneity between groups, applying parametric and non-parametric tests such as the Student's t-test and Fisher's exact test. To identify predictor variables potentially influencing the interlabial gap, a simple linear regression was performed for each cephalometric variable. Variables with a p-value &lt;0.20 were considered to have greater influence and were included in a second regression analysis using an exploratory overfitting approach to avoid excluding potentially relevant variables. Multiple linear regression models were constructed for each study group, including only the variables selected in the previous step, to determine which variables maintained a statistically significant association with the interlabial gap within each group. Finally, a global regression model including the entire sample was developed to identify significant predictors of interlabial gap for the whole population. Statistical significance was set at p&lt;0.05 for all analyses. All statistical analyses were conducted using SPSS version 26 for Windows (IBM SPSS, Chicago, IL).

## Results

Table 2 shows the matching process between the groups, indicating no significant differences in sex and age (p&gt;0.005).

Table 2

Cephalometric characteristics of both groups are shown in Table 3.

Table 3

Table 4 displays the general influence of all predictor variables on interlabial gap in millimetres, identifying those with possible significant influence (p&lt;0.200) for inclusion in the overfitting-based regression.

Table 4

Table 5 presents the regression results of selected predictor variables for each group. In the control group, significant influences were found for maxillomandibular divergence (B=0.03, p=0.019), upper lip height (B=0.06, p=0.032), upper lip to S-line distance (B=-0.05, p=0.024), and lower lip to S-line distance (B=0.05, p=0.047). In the AOB group, only lower facial height had a significant influence (B=0.31, p=0.047).

Table 5

Table 6 presents the final multiple linear regression for the entire sample, revealing that anterior open bite (B=0.703, p=0.009) and lower facial height (B=0.177, p=0.027) significantly influenced the interlabial gap.

Table 6

## Discussion

This study compared how known cephalometric measurements influence the interlabial gap in individuals with and without AOB, aiming to determine whether specific cephalometric traits increase this gap in AOB patients compared to controls. The two groups were matched for age and sex, ensuring no significant differences in these covariates. This matching enhances internal validity and minimizes selection bias. Moreover, the ANB angle, showed a statistically significant difference between the groups (p = 0.032). Thus, the control group exhibited a skeletal Class I relationship, while the AOB group displayed a tendency toward skeletal Class II, a pattern frequently associated with this malocclusion (21 - 24). However, despite the statistically significant difference, the ANB values were numerically close, and additionally this variable did not emerge as a significant predictor of the interlabial gap in the regression models. Several other cephalometric values differed significantly between the groups: SN-GoMe (p = 0.003), interincisal angle (p &lt; 0.001), upper incisor inclination (U1/NA, p &lt; 0.001), maxillomandibular divergence (p = 0.001), and AFH/PPH ratio (p = 0.006). These findings confirm that AOB is associated with a hyperdivergent skeletal pattern, as commonly described in the literature (21 - 25). Moreover, the study identified the most influential variables affecting the interlabial gap in both groups. In the control group, maxillomandibular divergence (p = 0.032), upper lip to S-line (p = 0.005), and lower lip to S-line (p = 0.012) were significant. These results suggest that, in individuals without AOB, the interlabial gap is primarily influenced by dentoalveolar and labial projection factors. These findings align with the research of Cunningham et al. (26) who emphasized the importance of perioral aesthetics in dental occlusion. In contrast, in the AOB group, only lower facial height had a significant influence (p = 0.047), indicating that among the various cephalometric differences observed in this group (including SN-GoMe, interincisal angle, U1/NA, maxillomandibular divergence, and AFH/PPH), the vertical dimension of the lower facial third is the key factor modifying the interlabial gap and determining lip seal. Therefore, to improve lip seal in AOB patients, orthodontic treatment should primarily focus on controlling the vertical dimension, such as by intruding the upper and lower molars. When analysing both groups in a combined regression model, the results confirmed that the presence of anterior open bite (p = 0.009) and increased lower facial height (p = 0.027) were the only significant predictors influencing the interlabial gap. This highlights the clinical relevance of these conditions and guides orthodontists to target both aspects to achieve adequate lip seal, aesthetics, and function. One limitation of the study is its cross-sectional design, which does not allow for causal inference. Additionally, functional variables such as muscle tone or oral habits (e.g., mouth breathing), which may influence lip seal, were not included and should be considered in future research.

## Conclusions

The interlabial gap in individuals with anterior open bite is mainly influenced by vertical facial dimension, while in controls, maxillomandibular divergence and lip-to-S-line distances play a greater role. Moreover, the presence of anterior open bite and increased vertical facial dimension are the most influential factors affecting interlabial gap, highlighting the need to address both in treatment planning.

## Figures and Tables

**Table 1:** Outlines the definitions of the cephalometric points and angles utilized in the study.

Angular measurements	Definition
SNA	Angle between the nasion-sella line and point A.
SNB	Angle between the nasion-sella line and point B.
ANB	Angle between points A and B.
SN-GoGn	Angle between the nasion-sella line and the mandibular plane.
1/1	Angle between the longitudinal axes of the upper and lower incisors.
U1/NA	Angle between the longitudinal axis of the upper incisor and the NA line.
L1/NB	Angle between the longitudinal axis of the lower incisor and the NB line.
Maxillomandibular divergence	Angle between the maxillary plane and the mandibular plane.
AFH/PPH	Angle between the anterior and posterior facial heights.
Linear measurements	Definition
Lower facial height	Distance in mm between ANS y Me points.
Upper molar height	Distance in mm from the mesiobuccal cusp of the 1st UM perpendicularly to the palatal plane.
Lower molar height	Distance in mm from the mesiobuccal cusp of the 1st LM perpendicularly to the mandibular plane.
Upper incisor distance to the palatal plane	Measurement in mm from the incisal edge of the upper incisor to the palatal plane.
Lower incisor distance to the mandibular plane	Measurement in mm from the incisal edge of the lower incisor to the mandibular plane.
Interlabial distance	Distance in mm between the upper and lower lip.
Upper lip height	Distance in mm from a line parallel to the Frankfurt plane passing through the subnasale to the tip of the upper stomion.
Upper lip position relative to Steiner's S-line	Distance in mm of the upper lip position relative to Steiner's S-line.
Lower lip position relative to Steiner's S-line	Distance in mm of the lower lip position relative to Steiner's S-line.

1

**Table 2:** Initial demographic characteristics in both evaluated groups.

Group		Sex			p-value
		Female	Male	Total	
Control	n	35	20	55	0.841*
	%	63.6	36.4	100	
Anterior open bite	n	37	18	55	
	%	67.3	32.7	100	
Total	n	72	38	110	
	%	65.5	34.5	100	
Group		Sex			
		n	Mean	SD	p-value
Control		55	27.27	6.41	0.325**
Anterior open bite		55	26.02	6.88	

* Fisher’s exact test, ** Student’s t-test

**Table 3:** Cephalometric characteristics in both evaluated groups.

Cephalometric measurement	Group	n	Mean	SD	p-value
SNA	Control	55	83.70	3.84	0.156
	Anterior open bite	55	82.68	3.61	
SNB	Control	55	79.51	3.61	0.761
	Anterior open bite	55	79.75	4.40	
ANB	Control	55	4.19	2.56	0.032*
	Anterior open bite	55	2.94	3.39	
SN-GoGn	Control	55	33.88	5.67	0.003*
	Anterior open bite	55	37.96	7.97	
Interincisal angle	Control	55	127.82	10.14	<0.001*
	Anterior open bite	55	119.52	12.02	
U1/NA	Control	55	20.13	7.01	<0.001*
	Anterior open bite	55	27.61	8.02	
L1/NB	Control	55	27.36	7.06	0.078
	Anterior open bite	55	29.93	8.09	
Maxillomandibular divergence	Control	55	25.28	5.37	0.001*
	Anterior open bite	55	29.19	6.08	
AFH/PPH	Control	55	66.32	6.25	0.006*
	Anterior open bite	55	63.17	5.44	
Lower facial height	Control	55	66.39	5.01	0.265
	Anterior open bite	55	67.50	5.45	
Upper molar height	Control	55	23.29	2.10	0.587
	Anterior open bite	55	23.51	2.13	
Lower molar height	Control	55	31.48	3.20	0.031*
	Anterior open bite	55	30.21	2.85	
U1/PP	Control	55	30.58	2.87	0.115
	Anterior open bite	55	31.51	3.29	
L1/PM	Control	55	40.74	4.68	0.152
	Anterior open bite	55	39.59	3.59	
Interlabial distance	Control	55	0.08	0.28	<0.001*
	Anterior open bite	55	1.19	1.62	
Upper lip height	Control	55	22.91	2.01	0.339
	Anterior open bite	55	22.48	2.64	
Upper lip to S-line	Control	55	-2.40	2.67	0.213
	Anterior open bite	55	-1.74	2.80	
Lower lip to S-line	Control	55	-1.34	2.56	0.001*
	Anterior open bite	55	0.47	2.85	

Student’s t-test, * Significant

**Table 4:** Influence of predictor variables on interlabial distance in millimeters.

Group	Predictor Variables	B	p-value	95.0% confidence Interval for B	
				Lower Limit	Upper Limit
Control	(Constant)	-0.85	0.803	-7666.00	5972.00
	Age	-0.01	0.282	-0.02	0.01
	Sex	-0.03	0.784	-0.23	0.18
	SNB	0.00	0.966	-0.04	0.04
	ANB	-0.03	0.490	-0.12	0.06
	SN-GoGn	-0.02	0.393	-0.07	0.03
	Interincisal angle	0.00	0.816	-0.03	0.04
	U1/NA	-0.01	0.671	-0.06	0.04
	L1/NB	0.01	0.433	-0.02	0.04
	DM	0.04	0.032	0.00	0.08
	AFH/PPH	-0.01	0.588	-0.03	0.02
	Lower facial height	-0.05	0.182	-0.11	0.02
	Upper molar height	0.05	0.151	-0.02	0.13
	Lower molar height	0.04	0.158	-0.02	0.10
	U1/PP	0.01	0.865	-0.07	0.08
	L1/PM	-0.01	0.303	-0.03	0.01
	Upper lip height	0.05	0.110	-0.01	0.11
	Upper lip to S-line	-0.09	0.005	-0.14	-0.03
	Lower lip to S-line	0.09	0.012	0.02	0.15
Anterior open bite	(Constant)	-17704.00	0.167	-43191.00	7783.00
	Age	-0.02	0.577	-0.10	0.06
	Sex	-0.75	0.309	-2226.00	0.73
	SNB	-0.43	0.922	-9136.00	8286.00
	ANB	-0.13	0.975	-8891.00	8622.00
	SN-GoGn	0.07	0.261	-0.06	0.21
	U1/NA	-0.02	0.684	-0.13	0.09
	L1/NB	-0.09	0.072	-0.19	0.01
	DM	-0.06	0.661	-0.31	0.20
	AFH/PPH	0.21	0.046	0.00	0.41
	Lower facial height	0.37	0.070	-0.03	0.77
	Upper molar height	-0.37	0.124	-0.85	0.11
	Lower molar height	-0.38	0.047	-0.75	0.00
	U1/PP	0.06	0.697	-0.26	0.38
	L1/PM	0.10	0.499	-0.20	0.39
	Upper lip height	-0.16	0.238	-0.42	0.11
	Upper lip to S-line	-0.07	0.655	-0.37	0.24
	Lower lip to S-line	0.08	0.584	-0.22	0.39
	SNA	0.44	0.920	-8284.00	9155.00

The highlighted values correspond to variables with a p-value < 0.200, which were considered for a new multiple linear regression analysis.

**Table 5:** Second multiple linear regression evaluating the influence of predictor variables on interlabial distance in millimeters in each evaluated group.

Group	Predictor Variables	B	p-value	95.0% Confidence Interval for B	
				Lower Limit	Upper Limit
Control	(Constant)	-1304.00	0.015	-2343.00	-0.27
	L1/NB	0.00	0.942	-0.01	0.01
	DM	0.03	0.019*	0.01	0.05
	Lower facial height	-0.04	0.116	-0.09	0.01
	Upper molar height	0.05	0.084	-0.01	0.11
	Lower molar height	0.03	0.302	-0.03	0.08
	Upper lip height	0.06	0.032*	0.01	0.11
	Upper lip to S-line	-0.05	0.024*	-0.10	-0.01
	Lower lip to S-line	0.05	0.047*	0.00	0.10
Anterior open bite	(Constant)	2456.00	0.487	-4590.00	9501.00
	L1/NB	-0.02	0.653	-0.09	0.06
	DM	-0.06	0.445	-0.21	0.09
	Lower facial height	0.31	0.047*	0.00	0.62
	Upper molar height	-0.31	0.155	-0.73	0.12
	Lower molar height	-0.26	0.124	-0.60	0.08
	Upper lip height	-0.21	0.111	-0.46	0.05
	Upper lip to S-line	0.12	0.411	-0.17	0.40
	Lower lip to S-line	0.03	0.822	-0.24	0.30

* Significant

**Table 6:** Multiple linear regression on the influence of predictor variables on interlabial distance in mm in both evaluated groups.

Predictor Variables	B	p-value	95.0% Confidence Interval for B	
			Lower Limit	Upper Limit
(Constant)	-0.321	0.854	-3781.000	3138.000
Presence of anterior open bite	0.703	0.009*	0.182	1224.000
L1/NB	-0.001	0.972	-0.036	0.035
Maxillomandibular divergence	-0.026	0.487	-0.098	0.047
Lower facial height	0.177	0.027*	0.021	0.334
Upper molar height	-0.117	0.231	-0.310	0.076
Lower molar height	-0.160	0.070	-0.333	0.013
Upper lip height	-0.126	0.079	-0.267	0.015
Upper lip to S-line	0.003	0.961	-0.135	0.142
Lower lip to S-line	0.043	0.565	-0.105	0.191

* Significant

## Data Availability

The data that supports the findings of this study are available from the corresponding author upon reasonable request.
